# Fast coalescence of metallic glass nanoparticles

**DOI:** 10.1038/s41467-019-13054-z

**Published:** 2019-11-20

**Authors:** Yuan Tian, Wei Jiao, Pan Liu, Shuangxi Song, Zhen Lu, Akihiko Hirata, Mingwei Chen

**Affiliations:** 1 Department of Materials Science and Engineering and Hopkins Extreme Materials Institute, Johns Hopkins University, Baltimore, MD 21218 USA; 2 State Key Laboratory of Metal Matrix Composites, School of Materials Science and Engineering, Shanghai Jiao Tong University, Shanghai, 200030 China; 3 Advanced Institute for Materials Research, Tohoku University, Sendai, 980-8577 Japan; 4 Mathematics for Advanced Materials-OIL, AIST-Tohoku University, Sendai, 980-8577 Japan

**Keywords:** Structure of solids and liquids, Nanoparticles, Glasses

## Abstract

The coarsening of crystalline nanoparticles, driven by reduction of surface energy, is the main factor behind the degeneration of their physical and chemical properties. The kinetic phenomenon has been well described by various models, such as Ostwald ripening and coalescence. However, the coarsening mechanisms of metallic glass nanoparticles (MGNs) remains largely unknown. Here we report atomic-scale observations on the coarsening kinetics of MGNs at high temperatures by in situ heating high-resolution transmission electron microscopy. The coarsening of the amorphous nanoparticles takes place by fast coalescence which is dominated by facet-free surface diffusion at a lower onset temperature. Atomic-scale observations and kinetic Monte Carlo simulations suggest that the high surface mobility and the structural isotropy of MGNs, originating from the disordered structure and unique supercooled liquid state, promote the fast coalescence of the amorphous nanoparticles at relatively lower temperatures.

## Abstract (web summary)

The coarsening of amorphous metallic nanoparticles remains poorly understood. Here, the authors combine high resolution microscopy and atomistic simulations to show the disordered structure of amorphous nanoparticles makes them coarsen faster than crystalline ones.

## Introduction

The disordered atomic structure endues metallic glasses (MGs) with many unique physical and chemical properties for structural and functional applications^1–4^. In general, the preparation of MGs requires rapid cooling by melt quenching or physical vapor deposition (PVD) to suppress competing crystallization^5,6^. Metallic glass nanoparticles (MGNs) with a small size and high surface-volume ratio are relatively easy to be synthesized by traditional powder metallurgy and sputtering and have attracted increasing attention for applications in additive manufacturing, composite reinforcement, catalysis, and biomedicine^7–12^. In these applications, nanoparticle coarsening is essential in the sintering kinetics and structural and functional retention. For its importance, the coarsening mechanisms of crystalline particles have been widely studied in past decades^13–17^. Typically, the mass transport process of particle coarsening takes place by four possible pathways: viscous flow, evaporation-condensation, volume diffusion, and surface diffusion^18^. These mechanisms show different kinetic behaviors of coalescence and significantly influence sintering rates^19^ and stability of particles^20^. In general, surface diffusion is the dominant mechanism for the coalescence of crystalline particles when their sizes are smaller than ∼1 μm and temperatures are above surface roughening points^21–23^. For macroscopic particles (>1 mm), the coarsening is usually accomplished by viscous flow regardless of crystalline and amorphous structures^24,25^. However, the coarsening kinetics and underlying mechanisms of MGNs at high temperatures remain largely unknown although fast surface dynamics of nanostructured MGs have widely been observed in both experiments^26,27^ and simulations^28^.

In this study, we employ micro-chip based ultra-stable heating stage, in combination with state-of-the-art aberration-corrected transmission electron microscopy (TEM) and highly-sensitive direct electron detector, to study high-temperature coalescence by at atomic-scale in situ observations. Our experiments reveal that the high-temperature coalescence of MGNs, dominated by facet-free surface diffusion, is much faster than that of crystalline particles, benefiting from the isotropic disordered structure of glasses and unique supercooled liquid state. The atomic observations and kinetic Monte Carlo (KMC) simulations demonstrate that the atomic structure of nanoparticles plays an important role in particle coalescence, in addition to the conventional wisdom of particle size and morphology.

## Results

### In situ TEM experiment setup

**Fig. 1:**
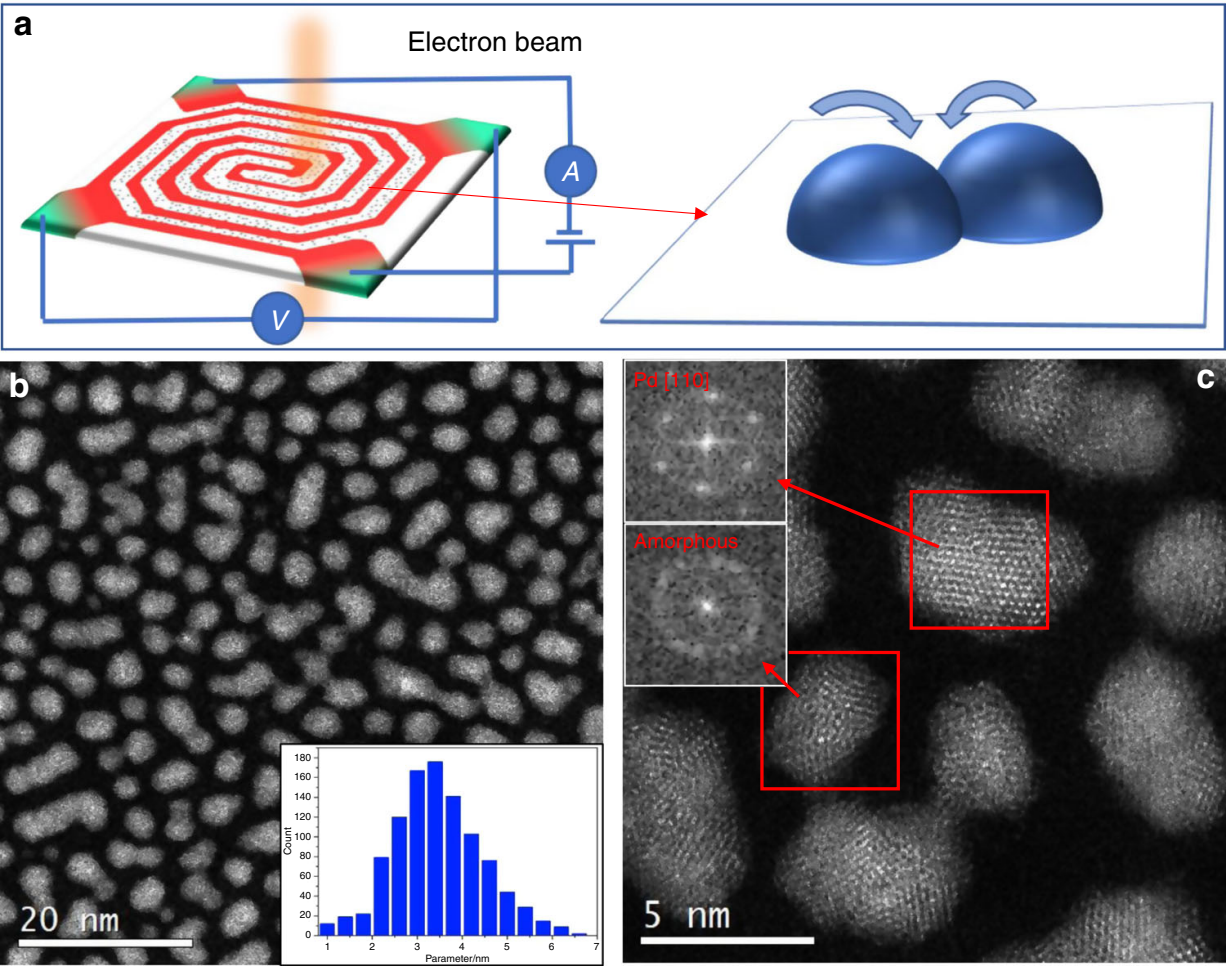
Experiment setup and TEM observation on the as-prepared sample. **a** Schematic diagrams showing the experimental setup for in situ TEM observations on particles sintering. Resistance wires on micro-chip enable precise control of temperature by electrical current. **b** Low-magnification HAADF-STEM image of the as-deposited PdSi nanoparticles on the observation window. The inset image shows the statistical size distribution of PdSi nanoparticles. **c** HAADF-TEM image of as-deposited PdSi nanoparticles, showing the co-existence of crystalline and amorphous nanoparticles

Figure 1a shows schematic diagrams of the in situ heating experimental setup for TEM observations. The heating micro-chip is comprised of patterned Pt wire circuits on 20 nm thick amorphous Si_3_N_4_ membrane which acts as the background-contrast-free support. The temperatures can be precisely controlled from room temperature up to 1273 K by tuning the voltage and current of the Pt circuits from an external power supplier. Pd_81_Si_19_ nanoparticles were uniformly deposited on the amorphous Si_3_N_4_ membrane by magnetron sputtering (Fig. 1b). The inset image in Fig. 1b shows the size distribution of the as-sputtered particles. These particles have an average size of ∼3.5 nm and most of them distribute in a narrow range between ∼2 and 5.5 nm. The structures of the as-deposited nanoparticles were investigated by high-resolution scanning TEM (STEM) with a high-angle annular dark field (HAADF) detector (Fig. 1c). Both crystalline and amorphous nanoparticles can be observed. The crystalline particles have a face-centered cubic (FCC) structure as determined by both HAADF-STEM image and the fast Fourier transform pattern (the inset of Fig. 1c). For the MGNs, short-range structure order is occasionally visible in the atomic images when local atomic clusters, most likely trigonal prisms^29,30^, have an in phase orientation. The co-existence of the crystalline and amorphous nanoparticles on one chip gives us a unique opportunity to observe the coalescence of individual amorphous-amorphous, crystal-crystal, and amorphous-crystal pairs under the same experimental conditions. As a generic tendency, the particles with a smaller size tend to be amorphous while larger particles are usually crystalline. The propensity that the amorphous structure remains in smaller particles is expected to associate with the critical nucleation size of the FCC crystal^31^, which depends on sputtering conditions, in particular the substrate temperatures (see the “Methods” section).

### In situ TEM observations on particle coalescence

**Fig. 2:**
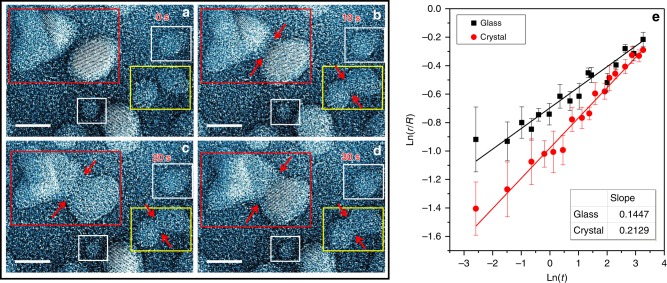
Coalescence of amorphous and crystalline nanoparticle pairs at 738 K. **a**–**d** Video of sequential images showing particles coalescence of amorphous PdSi particle pair (in yellow rectangle) and crystalline particle pair (in red rectangle) at 738 K. The stability of small particles in white rectangles demonstrates that the effect of Ostwald ripening effect is negligible. Scale bar: 5 nm. **e** The evolution of neck diameter as a function of time of crystalline-crystalline and amorphous-amorphous particles. The data are fitted using least-squares approximation, with the fitted slopes shown in the inset table. Error bar represents standard deviation of 12 measurements of the neck width in corresponding frame

In the in situ experiments, the sample was slowly heated from room to high temperatures. When the sample’s temperature is close to the glass transition point (*T*_g_, ~640 K) of the alloy, the as-sputtered MGNs start to relax to a more spherical shape (Supplementary Movie 9). The morphology changes, driven by surface tension, indicate that the sputtered sample is comprised of individual nanoparticles, not a continuous film, and the interaction between the particles and amorphous Si_3_N_4_ substrate is trivial. The sequential video images show the coalescence process of glassy and crystalline particles at 738 K, which is ∼98 K above *T*_g_ and 482 K below the melting point (*T*_m_) of the alloy (Fig. 2a–d, Supplementary Figs. 3 and 4 and the Supplementary Movie 1). The *T*_g_ value was measured from Pd_81_Si_19_ glass ribbons using differential scanning calorimetry (DSC) (the Supplementary Fig. 1). The kinetic transition temperature *T*_g_ of the nanoparticles may be slightly different from the ribbon samples but should not have a large variation because the weak dependence of *T*_g_ on sample sizes^32^. Since the observation temperature is above *T*_g_, the MGNs are in the dynamically equilibrium supercooled liquid state. Thus, excess defects, such as free volumes, generated by the sample preparation will be eliminated and should not affect the coalescence kinetics of the nanoparticles. The isolated amorphous particles, regardless of large or small sizes, in the white rectangles (Fig. 2a–d) show marginal size changes at the observation temperature, implying that Ostwald ripening (monomer attachment) is negligible for the coarsening of amorphous nanoparticles as the temperature may not be high enough for noticeable long-range diffusion in the vacuum environment. In contrast, the coarsening by coalescence takes place for both crystalline and amorphous nanoparticles. As examples, a pair of large particles (*d*_1_ ~ 7.17 nm, *d*_2_ ~ 7.79 nm) is displayed in the red rectangle and a pair of small particles (*d*_1_ ~ 3.74 nm, *d*_2_ ~ 3.92 nm) in the yellow rectangle (Fig. 2a–d). The lattice fringes shown in the large particles demonstrate their crystalline nature, while the smooth spherical shape without any visible lattice fringe of the small particles evidences the amorphous state. Initially, a gap (0.5–0.8 nm) between the particles is visible for both crystalline and glassy pairs. After about 10 s, the two particles in each pair become contacted by forming a narrow contact neck. The coalescence continues by gradually increasing the diameter of the contact necks as shown in Fig. 2c, d. The disordered atomic structure of the amorphous particles remains unchanged during the high-temperature morphology evolution. It is worth noting that the observation temperature is far above the crystallization temperature (∼665 K) of the Pd_81_Si_19_ glass measured from ribbon samples (see the Supplementary Fig. 1). The abnormal thermal stability against crystallization indicates that the size of the coarsened amorphous nanoparticles may be smaller than the critical nucleation size of the FCC crystal at the high temperature (See the “Methods” section), or the nano-scale sizes of the MGNs stabilize the supercooled liquid state against crystallization^33^.

The kinetics of the particle coalescence can be quantitatively described by measuring the evolution of contact neck radius with the assumption that neck growth is fulfilled by consuming the atoms from the paired particles. It has long been known that the kinetics of neck growth with time can be described by a power law relation^18,24^:

1 $$r^n = Kt$$

where *r* is the neck radius, *t* is time, *K* is a constant which depends on the average radius of particles, temperature, atomic volume, surface energy and diffusivity of materials. The exponent *n* is a characteristic variable associated with the mass transport mechanisms. Although the power law is derived based on classic continuum model, it has been commonly applied on the nanoparticle coalescence^20,21^ since dimensionless continuum theory is valid in nanoscale if the isotropic surface tension can be reserved. In general, *n* = 5 (*r*^5^ ∞ *t*) indicates that the coalescence is conducted by volume diffusion^24^ or grain boundary diffusion^18^ while *n* = 7 (*r*^7^ ∞ *t*) suggests a surface diffusion controlled dynamic process, as suggested by Kuczynski^24^. On the basis of the in situ TEM observations, we plotted the evolution of neck diameter with time in natural logarithm for the glassy and crystalline pairs and fitted the data using the least-squares approximation (Fig. 2e). In these plots, the coalescence range spans from *r* = 0 to *r*_initial_ (initial paired particle size). Both crystalline and glassy pairs show a linear dependence of the neck radius changes on the logarithm of time, but have different slopes, i.e., *1/n*. The coalescence of crystalline particles has a slope of 0.2129 ± 0.0097, between 1/4 and 1/5, while the coalescence of amorphous particles has the slope of 0.1447 ± 0.0071, close to 1/7. The obvious difference in the slopes demonstrates that the atomic structure of the particles significantly influences the coalescence kinetics. Apparently, the coalescence of MGNs is dominated by surface diffusion, in line with the classical coalescence theory of two spherical particles with isotropic surface tension^26,34^. In contrast, the coalescence of crystalline particles appears to be influenced by a facet mediated surface diffusion, which can increase the exponent *n* from 1/7 up to ∼1/3 as suggested by KMC simulations^20,35^.

**Fig. 3:**
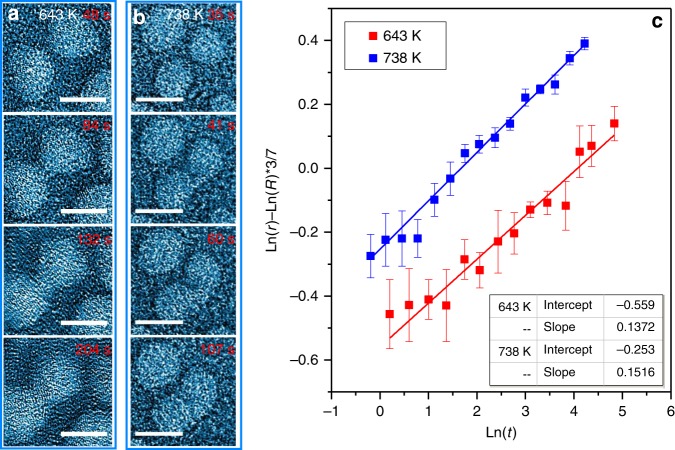
Coalescence of amorphous PdSi particles at different temperatures. **a**, **b** Video images showing the coalescence of particles at 643 and 738 K, respectively. Scale bar: 5 nm. **c** The evolution of neck diameter as a function of time in logarithm. The data are fitted using least-squares approximation, with the fitted data shown in the inset table. Error bar represents standard deviation of 12 measurements of the neck width in corresponding frame

The coalescence of the amorphous and crystalline nanoparticles was further investigated by in situ TEM observations at a lower temperature of 643 K, i.e., ∼3 K above *T*_g_ and ∼0.53 *T*_m_, which is below the surface roughening temperature of the FCC crystals (above ∼0.6 *T*_m_)^36,37^. The disordered atomic arrangement in the nanoparticles demonstrates their amorphous structure, as shown in Fig. 3a, b. The sequential HRTEM images of the amorphous particle coalescence at 643 K are shown in Fig. 3a (see Supplementary Fig. 5 and Supplementary Movie 2). Similar to the coalescence at the higher temperature of 738 K, the morphology evolution of the amorphous nanoparticle pair starts from the contact of two separate particles and then fulfills by gradually increasing the contact neck radius before the two small particles completely merge as a large one. For comparison, another set of sequential HRTEM images of the amorphous particle coalescence at 738 K are shown in Fig. 3b (see Supplementary Fig. 6 and Supplementary Movie 3). The 95 K lower sintering temperature obviously slows down the coalescence kinetics of the amorphous particles and necessitates much longer time for the particle coarsening. However, the coalescence slopes at 643 and 738 K are nearly parallel to each other and have the similar slopes of 0.1373 ± 0.0086 and 0.1517 ± 0.0053, respectively, which are all very close to 1/7 (Fig. 3c). This consistency further demonstrates that surface diffusion remains as the controlling factor of the MGN coalescence of at the low and high temperatures in the supercooled liquid region. In contrast, coalescence of crystalline nanoparticle pairs cannot be found at the low temperature during our in situ TEM observations. This may be due to the fact that the temperature is too low to activate extensive long-distance diffusion events on crystal surfaces at the observation time scale.

The lower activation temperatures and faster surface diffusion of MGNs are further demonstrated by the coalescence behavior of asymmetric amorphous-crystal pairs. When a crystalline particle pairs with an amorphous one that usually has a smaller size, the coalescence of the asymmetric amorphous-crystal pairs is dominated by the diffusion from the amorphous particles to the crystalline one. As a result, the amorphous nanoparticles gradually merge into the crystals (see the Supplementary Fig. 2, Supplementary Movies 4 and 5). These observations further demonstrate that the amorphous structure, most likely associated with the supercooled liquid state and isotropic disordered structure, promotes the surface diffusion and coalescence at relatively lower onset temperatures at which the crystalline nanoparticles are still stable.

For surface diffusion driving coalescence, the change of contact neck radius with time is given by^38^:

2 $$\frac{{r^7}}{{R^3}} = \frac{{56V_{\mathrm{a}}\gamma _{\mathrm{s}}D_{\mathrm{s}}\delta _{\mathrm{s}}t}}{{kT}}$$

where *r* is the neck radius, *R* is the particle radius, *T* is the temperature, V_a_ is the atomic volume, *γ*_s_ is the surface energy, *D*_s_ is the surface diffusivity, *δ*_s_ is the surface diffusive width and *k* is Boltzmann constant. The dependence of the surface diffusivity *D*_s_ on temperature is described by the equation^39^:

3 $$D_{\mathrm{s}} = D_{{\mathrm{s}},0}e^{ - \Delta G/kT}$$

in which *D*_s,0_ is constant and Δ*G* is the activation energy of diffusion. The coalescence activation energy of the amorphous particles can be derived from $\left( {Ln\left( r \right) - \frac{3}{7}Ln\left( R \right)} \right)$ vs. *Ln*(*t*) plots based on two independent experiments at different temperatures (Fig. 3a, b). From the temperature dependence of intercepts of the two plots in Fig. 3c, the activation energy of surface diffusivity is estimated as Δ*G*_s_ ~ 0.97 eV (for the details, see “Methods”), which is close to the surface diffusion activation energy of Pd in a PdCuNiP metallic glass^40^.

In general, surface diffusivity is faster than volume diffusivity in both crystals^39^ and glasses^26,41,42^ due to lower energy barrier of diffusion. In this study, the activation energy of surface diffusion for the Pd_81_Si_19_ glass is measured to be Δ*G*_s_ ~ 0.97 eV, which is about half of that of the volume diffusion (1.7 eV) measured from the glassy alloy with a similar composition of Pd_77.5_Cu_6_Si_16.5_^43^. Interestingly, the coalescence kinetics of crystalline and amorphous nanoparticles with a similar size and the same composition (thus the same melting temperature) are quite different as revealed by the in situ TEM observations. Apparently, the atomic structure, order and disorder, of the nanoparticles plays a crucial role in the underlying coalescence kinetics, in addition to size and morphology of particles. The influence of the amorphous structure on the sintering kinetics could come from two aspects. First, the unique supercooled liquid state of glasses offers the glassy nanoparticles high atomic mobility at relative lower temperature, which could significantly decrease the onset temperature for activating the extensive surface diffusion^26,27,34^. Second, the disordered surface structure of the glassy nanoparticles effectively avoids the additional energy barriers from surface faceting of crystalline nanoparticles. In fact, it has been noticed that the coalescence kinetics of crystalline nanoparticles often does not follow the Kuczynski’s model of surface diffusion, *r*^7^ ∞ *t*, and have a smaller coalescence exponential, *r*^3^ ∞ *t*^20,35^. Since *n* = 3 has been obtained from crystalline particles with various sizes, the particle size is apparently not the key reason leading to the smaller *n* value. While, the surface faceting of crystalline nanoparticles has been suggested as the underlying mechanism of the failure of Kuczynski’s model in nano-regime—as the faceted surfaces are no longer isotropic.

### KMC simulations on particle coalescence

**Fig. 4:**
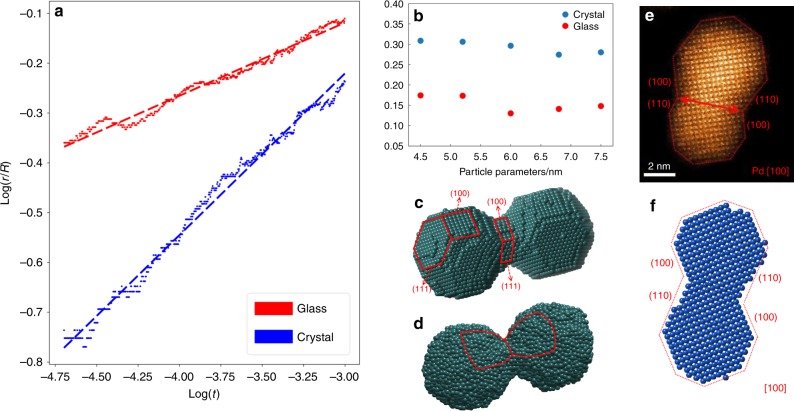
KMC simulations on the coalescence of crystalline and amorphous particle pairs. **a** KMC simulations on the evolution of neck diameters as a function of time in logarithm, at the simulation temperature of 400 K. **b** Coalescence slopes as a function of particle sizes. **c** Snapshots of a crystalline particle pair during coalescence in KMC simulation. The faceting takes place during the coalescence of the crystalline particles and due to the slow kinetics with a smaller exponential of ~3. **d** KMC snapshot of an amorphous particle pair during coalescence. Isotropic surface structure remains during the coalescence. **e** High resolution HAADF-STEM image of a faceted FCC particle pair at 738 K. **f** KMC snapshot of a FCC particle pair during coalescence, which shows similar facet behavior as in (**e**)

To understand the difference in high-temperature coalescence kinetics between crystalline and amorphous nanoparticles, we conducted KMC simulations (Fig. 4). The kinetic processes of the crystalline and amorphous nanoparticles are illustrated in the supplementary Movies 6 and 7, respectively. The evolution of neck diameters with time in logarithm for the glassy and crystalline pairs is plotted in Fig. 4a. Consistent with our in situ TEM observations, the coalescence between amorphous particles shows faster kinetics than crystalline ones. The plots of both crystalline and amorphous pairs can be well fitted by the power law relation, *r*^*n*^ = *Kt*. Again, the amorphous and crystalline particles have different *n* values of 6.80 and 2.96, respectively, consistent with the in situ TEM experiments. The snapshot of the simulated coalescence process of crystalline particles (Fig. 4c) shows the formation of {111} facets, marked by red lines, in the contact neck region between two particles. We noticed that the most frequent facet planes are {111}, {110} and {100}, in line with the surface energies of FCC crystals. The faceting of the crystalline particles during coalescence has also been caught by our in situ TEM observations as shown in Fig. 4e, in which {100} and {110} facets are formed at the contact neck region during coalescence of a crystalline pair at 738 K. This scenario can be well reproduced by the KMC simulation (Fig. 4f). Since mismatch angles between paired crystal particles may influence the faceting and coalescence kinetics, we systematically tilt the orientation of one crystal particle in a pair. The crystallographic mismatch introduces the formation of a grain boundary and misaligned facets between two particles. As the change in orientation partially impairs the formation of perfect facet in the contact neck region (shown in Supplementary Movie S8), the exponential in *r*^*n*^ = *Kt* relation falls into a range between 3 and 5 but still lowers than 7 for amorphous particles (Supplementary Fig. 7). The KMC snapshot on the amorphous particle coalescence (Fig. 4d) shows that the amorphous particles always keep a spherical shape with a smoothly curved surface in the contact neck region, indicating that isotropic surface tension and structure homogeneity persist during the coarsening. We also simulated the coalescence kinetics of amorphous and crystalline particle pairs with the particle sizes ranging from 4.5 to 7.5 nm. The exponentials in the *r*^*n*^ = *Kt* relation shows no obvious change with respect to particle size for both glassy and crystalline particles, as shown in Fig. 4b. This indicates that the significant difference in coalescence kinetics between glassy and crystalline particles is not caused by the trivial size difference. Moreover, surface segregation on the amorphous and crystalline particles cannot be detected by our atomic-scale observations. It was also reported that the surface segregation is negligible in this alloy system^44^. Since the same parameters (temperature, bond energy and attempt frequency) are used for amorphous and crystalline particles in the KMC simulations, surface isotropy and atomic structure are the only factors that make amorphous particles different from crystalline ones in the coalescence kinetics.

## Discussion

As revealed by our in situ TEM observations and KMC simulations, the coalescence of the amorphous nanoparticles can be well described by Kuczynski’s model of surface diffusion, *r*^7^ ∞ *t*, demonstrating that the continuum model is valid down to the length scale of several nanometers when the isotropic surface is reserved by the homogeneous amorphous structure. The facet-free surface diffusion could be one of the structural origins of fast surface diffusion and fast surface dynamics of nanostructured amorphous materials, which have been widely observed from MGs and organic glasses^34,40,42^. The surface chemical potential positively depends on the local curvature according to the Gibbs-Thompson relation:

4 $$\mu \left( r \right) = \mu ^\infty + \frac{{2\gamma \Omega }}{r}$$

where *μ*(*r*) is the chemical potential, *μ*^∞^ is the chemical potential of a flat surface, *γ* is surface energy, Ω is molar volume and the curvature is 1/*r*. Naturally, the formation of facets results in a lower chemical potential of in-plane atoms since *r* = +∞ for the flat facets in comparison with those of the atoms sitting on curved surfaces. Therefore, the facet tendency of the contact necks impairs the driving force of nanoparticle coalescence and, as a result, crystalline particle sintering is inevitably decelerated, while the MGNs are free from this effect. Moreover, the faceted particles are kinetically stabilized because the nucleation of new layers of atoms on facet is more difficult than on spherical surface^20,45,46^. Therefore, the agglomeration of MGNs would proceed faster than that of crystalline particles and thus are kinetically more suitable to facilitate the low-temperature fast sintering for additive manufacturing of MGs.

In conclusion, we found that the coalescence of MGNs is dominated by facet-free surface diffusion and shows faster kinetics and lower onset temperatures than those of crystalline counterparts. The finding of the atomic structure effect on surface diffusion and coalescence advances our understanding on coarsening kinetics of nanoparticles and surface dynamics of glasses. The fast coalescence, facilitated by the homogeneous amorphous structure and unique supercooled liquid state of MGs, also has important implications in developing low-temperature and fast additive manufacturing.

## Methods

### Sample preparation and DSC test

The Pd_81_Si_19_ alloy is chosen for the in situ heating TEM experiments for its good glass forming ability, high resistance to oxidation and high stability in vacuum^27,31,47,48^. The Pd_81_Si_19_ glass target was prepared by melting with the pure Pd and Si elements with the purity of 99.999%. PdSi nanoparticles were directly deposited onto the Si_3_N_4_ matrix of the micro-chip by radio frequency magnetic sputtering with the power of 50 W. The working argon pressure is set as 0.3 Pa and the substrate temperature is close to room temperature. The thermal properties of Pd_81_Si_19_ glass ribbons, prepared by melt spinning, were measured by Perkin-Elmer 8500 Differential Scanning Calorimetry (DSC) instrument at a heating rate of 40 K per minute with flowing pure Ar gas for protecting samples from possible oxidation.

### In situ heating TEM

The TEM and STEM were conducted on a JEOL JEM-2100F electron microscope equipped with double spherical aberration correctors for imaging and probing. The microscope was operated at 200 kV. High-angle annular dark-field scanning transmission electron microscopy (HAADF-STEM) images were taken using an annular-type detector with a collection angle ranging from 100 to 267 mrad. The in situ heating experiment was also performed using a DENS solutions heating holder, which provides temperature stability of 0.1 K and temperature accuracy of 2%. The TEM chamber pressure during in situ experiment is ~1.5 × 10^−5^ Pa. The high-resolution TEM video was recorded by Direct Detection Device (DDD) Camera DE-12 at a speed of 20 frames per second. To avoid possible electron beam damage and heating effect, the in situ observations were conducted at a low dose mode and the dose rate is less than 15 electrons pixel^−1^ frame^−1^. To reduce the accumulative heating effect from electron radiation on the sample, the observations on the same region was kept less than 5 min. The changes of the contact neck diameters were measured from each frame based on the change of contrast.

### Kinetic Monte Carlo simulations

The kinetic Monte Carlo simulations in this study follows the method in ref. ^35^. The simulation is conducted with the bond counting algorithm^49,50^ by python. The bond energy between different atoms is set as *E*_0_ = 0.1 eV, as used extensively in previous research^35^. The jump rate of single atom is defined by:

5 $$P = \nu \,{\mathrm{exp}}( - E_{\mathrm{A}}/k_{\mathrm{b}}T)$$

where *E*_A_ is the activation energy and the attempt frequency is $\nu = \frac{{k_{\mathrm{b}}T}}{h}$. Activation energy is defined as *E*_A_ = *nE*_0_, where *E*_0_ is bond energy, and *n* is bond number, i.e., first coordinate number. Only coordinate number is considered in the calculation of activation energy while the hoping distances are not counted. This is because the contribution of the hopping distance to the activation energy is much smaller (~10%) in comparison with that from coordinate number. The spatial fluctuation of coordinate number determines the mobility of atoms and provides the driving force of coalescence. The temperature is taken as 400 K to suppress atom desorption. The time increment is taken as $\Delta t = - \frac{{\ln \left( u \right)}}{k}$, where *k* is the rate constant for shifting out of a state and *u* is a random number. Pd FCC structure is taken as the atomic structure of the crystalline particles, as characterized in the experiment. While Molecular Dynamics (MD) simulation is used to construct amorphous structure for the amorphous particles. Since the mobility at the surface of a glass particle exceeds that of the bulk diffusion by more than 6 orders of magnitude^28^, the internal structure of amorphous particles is assumed to be stable during the coalescence process. In the KMC simulations, atoms jump at the well-defined FCC lattices for crystalline particles, and on the pre-calculated amorphous structure mesh-grids for amorphous particles. The diameters of crystalline particles and amorphous particles are set as 7.5 and 4.5 nm respectively to reproduce the condition in the in situ TEM experiment observations. The physical picture of this method is that the evolution of morphology proceeds by a set of atomic jumping events in which atoms jump to adjacent vacant atomic position with different rates that are determined by the energy barrier for the jumping events, i.e., the total bond energy of that atom. Final state of every jumping event has no effect on the jump probability.

### Estimation on surface diffusion activation energy

Applying natural logarithm processing on both side of the Eq. (2) by ref. ^38^:

6 $$r^7 = \frac{{56V_{\mathrm{a}}R^3\gamma _{\mathrm{s}}D_{\mathrm{s}}\delta _{\mathrm{s}}t}}{{kT}}$$

then, we get:

7 $${\mathrm{Ln}}\left( r \right) - \frac{3}{7}{\mathrm{Ln}}\left( R \right) = \frac{1}{7}{\mathrm{Ln}}\left( t \right) + \frac{1}{7}\left[ {{\mathrm{Ln}}\left( {\frac{{56V_{\mathrm{a}}\gamma _{\mathrm{s}}D_{\mathrm{s}}\delta _{\mathrm{s}}}}{k}} \right) + {\mathrm{Ln}}\left( {\frac{{D_{\mathrm{s}}}}{T}} \right)} \right]$$

Plug $D_s = D_{s,0}e^{ - \Delta G/kT}$ into previous one, we get:

8 $${\mathrm{Ln}}\left( r \right) - \frac{3}{7}{\mathrm{Ln}}\left( R \right) = \frac{1}{7}{\mathrm{Ln}}\left( t \right) + \frac{1}{7}\left[ {{\mathrm{Ln}}\left( {\frac{{56V_{\mathrm{a}}\gamma _{\mathrm{s}}D_{{\mathrm{s}},0}\delta _{\mathrm{s}}}}{k}} \right) - \frac{{\Delta G}}{{kT}} - {\mathrm{Ln}}\left( T \right)} \right]$$

Since, in the plot of Fig. 3c, the term on the left side of the equation is the y axis, and *Ln*(*t*) is the x axis, the rest of the term on the right side, $\frac{1}{7}\left[ {{\mathrm{Ln}}\left( {\frac{{56V_{\mathrm{a}}\gamma _{\mathrm{s}}D_{{\mathrm{s}},0}\delta _{\mathrm{s}}}}{k}} \right) - \frac{{\Delta G}}{{kT}} - {\mathrm{Ln}}\left( T \right)} \right]$, is represented by the y-intercept of the linear fitting. Here we have the coalescence data of amorphous particles at 643 K and 738 K, as shown in Fig. 3a, b. The average radius R of the sintered amorphous particles in Fig. 3a, b are respectively determined to be ~2.55 and ~1.95 nm. We assume that the particles have the same density, surface layer thickness and surface energy. Then, $- \frac{1}{7}\left( {\frac{{\Delta G}}{{kT}} + {\mathrm{Ln}}T} \right)$ changes the intercept in Fig. 3c of the curves for different temperatures, −0.559 ± 0.027 for *T* = 643 K and −0.253 ± 0.017 for *T* = 738 K respectively, by which we can estimate the Δ*G* value:

$$\frac{1}{7}\left( { - \frac{{\Delta G}}{{kT}} - {\mathrm{Ln}}T} \right)|_{T = 643{\mathrm{K}}}^{T = 738{\mathrm{K}}} = {\mathrm{intercept}}|_{T = 643{\mathrm{K}}}^{T = 738{\mathrm{K}}} = 0.559 - 0.253 = 0.306 \pm 0.044$$

thus:

$$\Delta G \approx 0.97\,{\mathrm{eV}} \pm 0.13\,{\mathrm{eV}}$$

### Estimation on the critical nucleation size

The dependence of critical nucleation size on temperature follows the Gibbs-Thomson equation^31,51^:

9 $$T_{\mathrm{d}} = T_\infty \left( {1 - \frac{{4\gamma }}{{dH_{\mathrm{f}}\rho _{\mathrm{s}}}}} \right)$$

where *T*_d_ is amorphization temperature for a spherical crystal with a diameter of *d*, *T*_∞_ is the melting temperature of the bulk sample, *γ* is the interfacial energy, *H*_f_ is the enthalpy of fusion, and *ρ*_s_ is density of the crystalline material. It has been reported that the critical nucleation size of Pd_80_Si_20_ nanoparticle is ~2.4 nm at room temperature^31^. As the melting temperature of Pd_81_Si_19_ is 1220 K according to the phase diagram^52^, we can estimate the critical nucleation size of Pd_81_Si_19_ nanoparticle at 738 K using Gibbs-Thomson equation: *d* = 4.61 nm. This accounts for the amorphous structure of small particles at the temperature higher than crystallization temperature *T*_x_.

## Supplementary information

Supplementary Information

Spplementary Movie 1

Spplementary Movie 2

Spplementary Movie 3

Spplementary Movie 4

Spplementary Movie 5

Spplementary Movie 6

Spplementary Movie 7

Spplementary Movie 8

Spplementary Movie 9

## Data Availability

Data and Python source code for the KMC simulations are available via request to the corresponding author.
