# Supplementary material for: Fast coalescence of metallic glass nanoparticles
Source: Nat Commun. 2019 Nov 20;10:5249. doi: 10.1038/s41467-019-13054-z (PMC6868181; doi:10.1038/s41467-019-13054-z)
Supplement: Supplementary file 1 — Supplementary Information [file 41467_2019_13054_MOESM1_ESM.pdf]

Supplementary information for

Fast Coalescence of Metallic Glass Nanoparticles

Tian et al.

Supplementary Figure 1-7

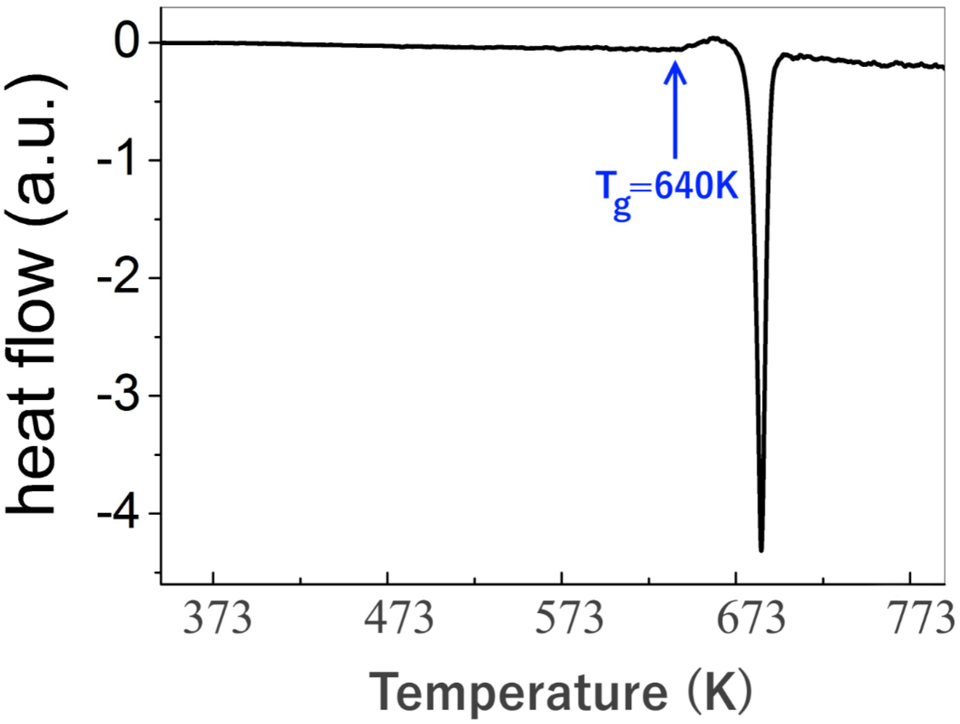

Supplementary Figure 1. DSC curve of $Pd_{81}Si_{19}$ taken from melt-spun ribbons at the scan rate of 40K/s. The glass transformation temperature is shown to be 640K.

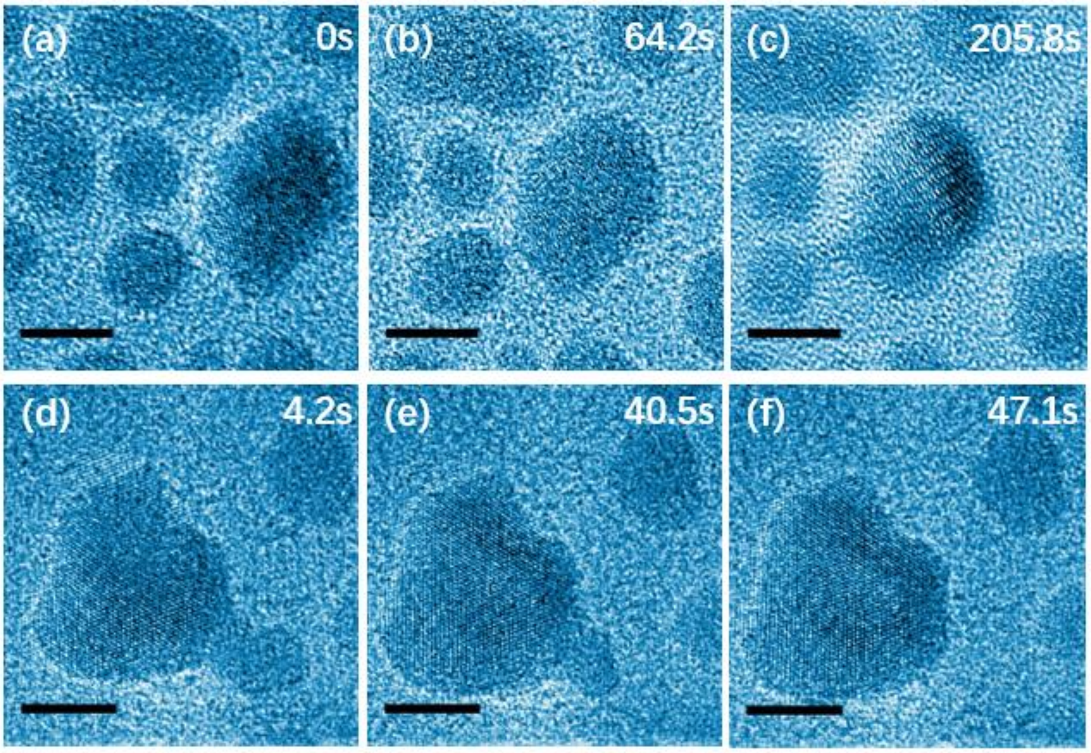

Supplementary Figure 2. Video sequential images showing the asymmetric coalescence between large crystalline particles and small amorphous particles at (a-c) 643K; and (d-f) 738K. Scale bar: 5 nm.

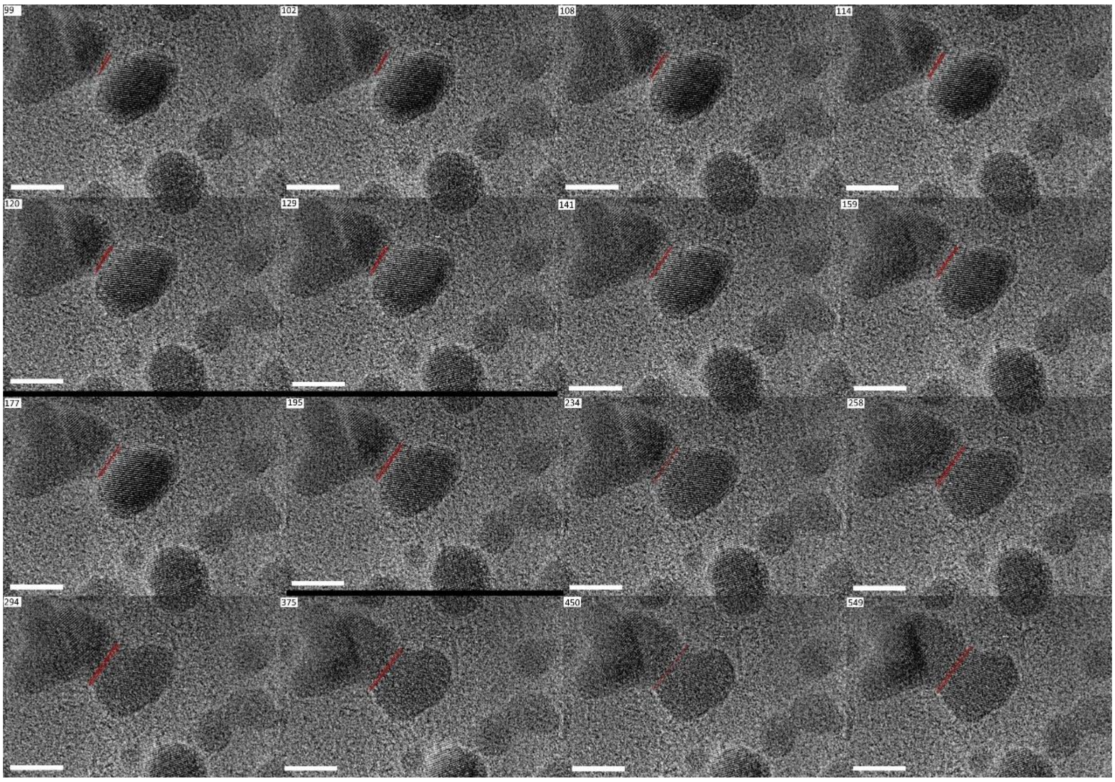

Supplementary Figure 3. Video sequential images from Movie 1 showing the measurements on the neck radius evolution of a crystal particle pair at 738K. The frame numbers are marked on the upper left of corresponding images. Scale bar: 5 nm.

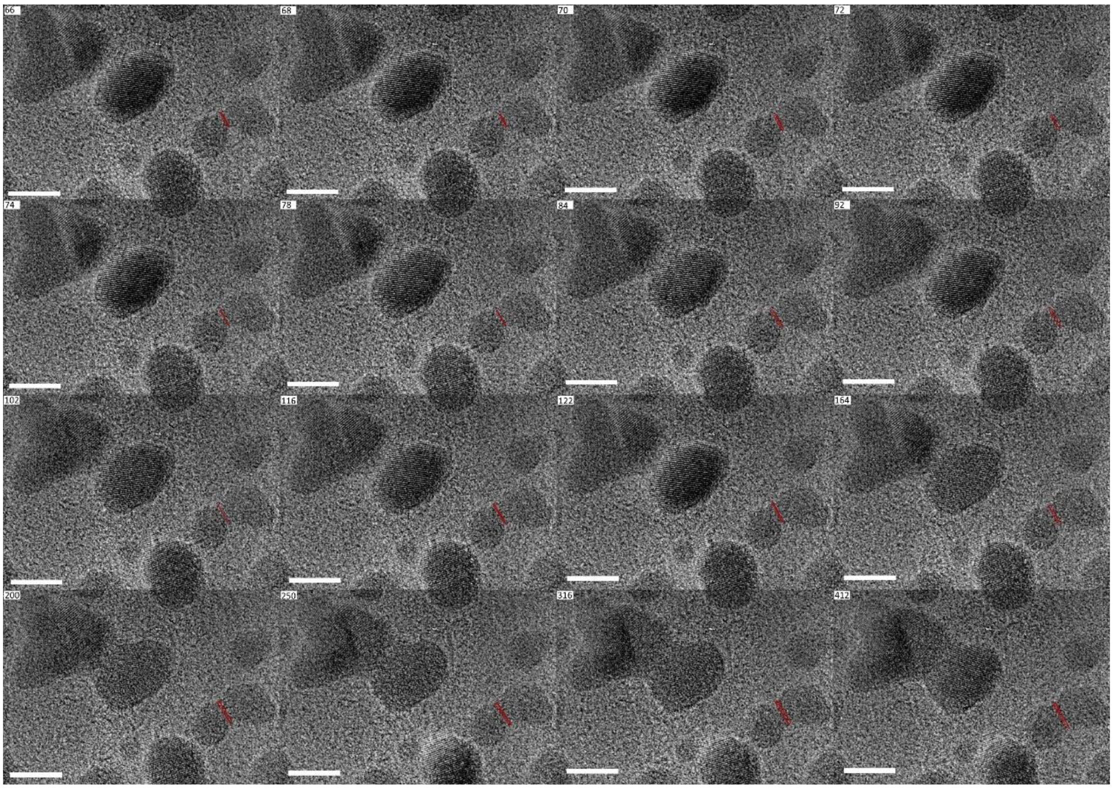

Supplementary Figure 4. Video sequential images from Movie 1 showing the measurements on the neck radius evolution of an amorphous particle pair at 738K. The frame numbers are marked on the upper left of corresponding images. Scale bar: 5 nm.

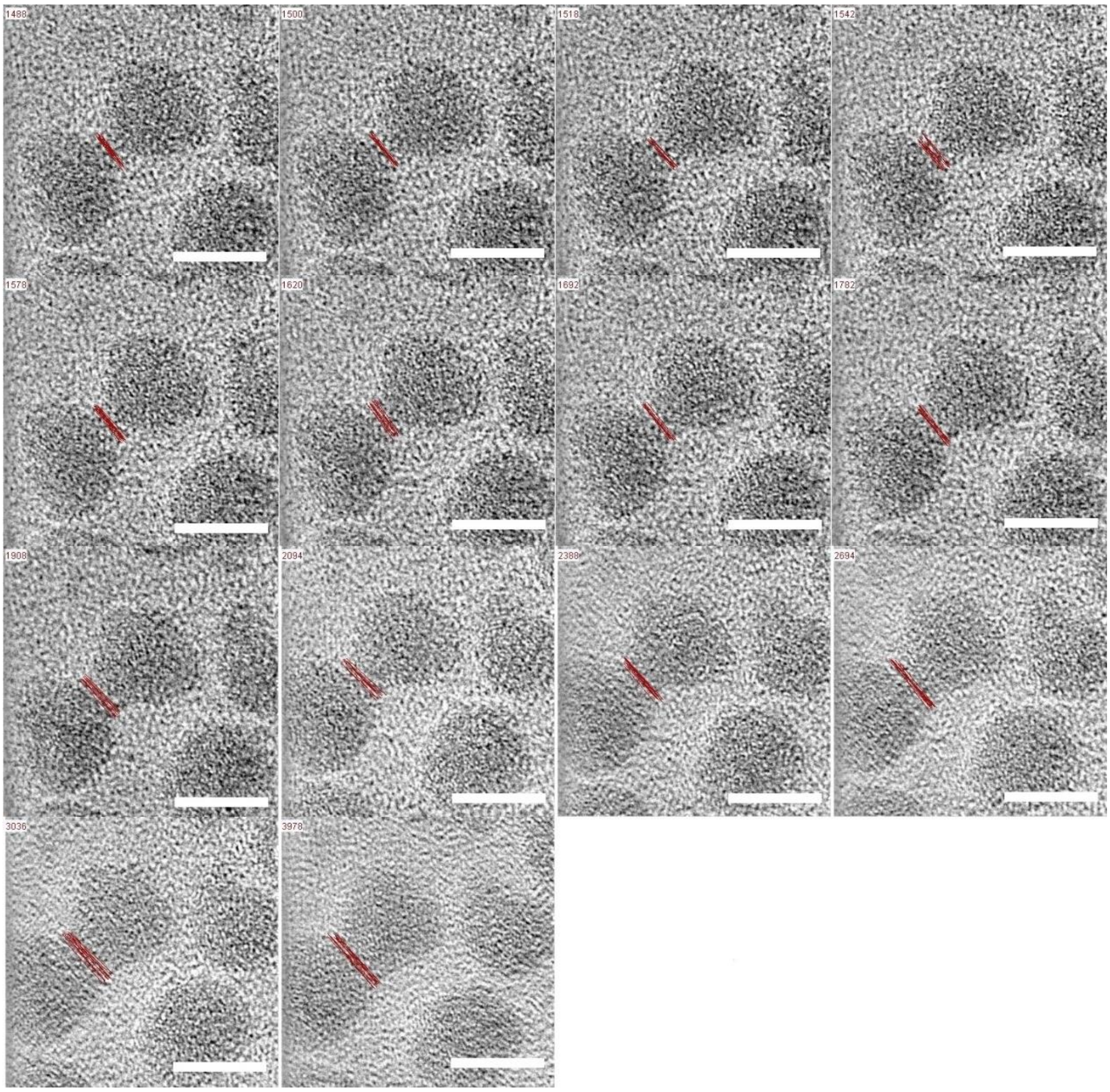

Supplementary Figure 5. Video sequential images from Movie 2 showing the measurements on the neck radius evolution of an amorphous particle pair at 643 K. The frame numbers are marked on the upper left of corresponding images. Scale bar: 5 nm.

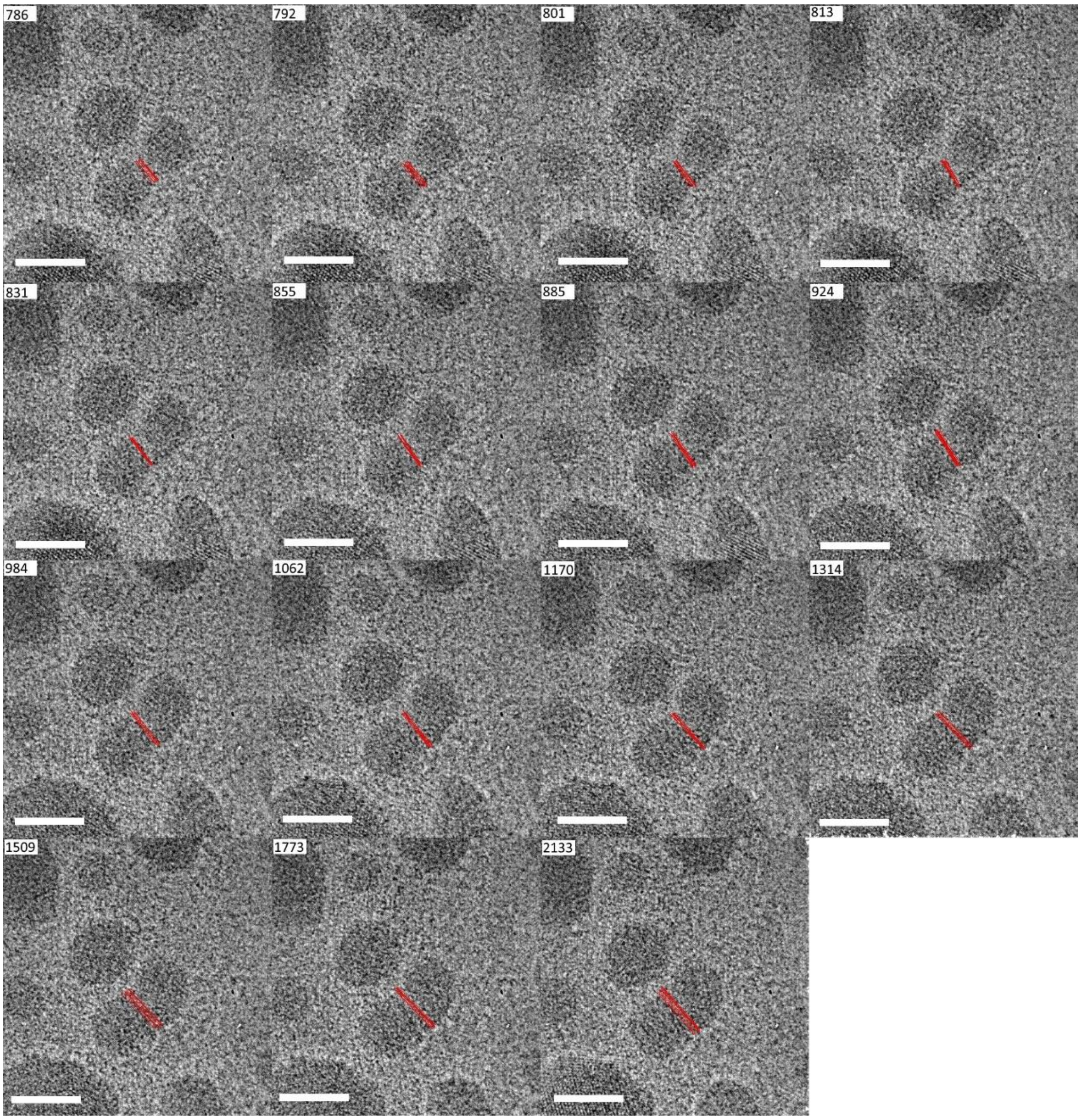

Supplementary Figure 6. Video sequential images from Movie 3 showing the measurements on the neck radius evolution of an amorphous particle pair at 738 K. The frame numbers are marked on the upper left of corresponding images. Scale bar: 5 nm.

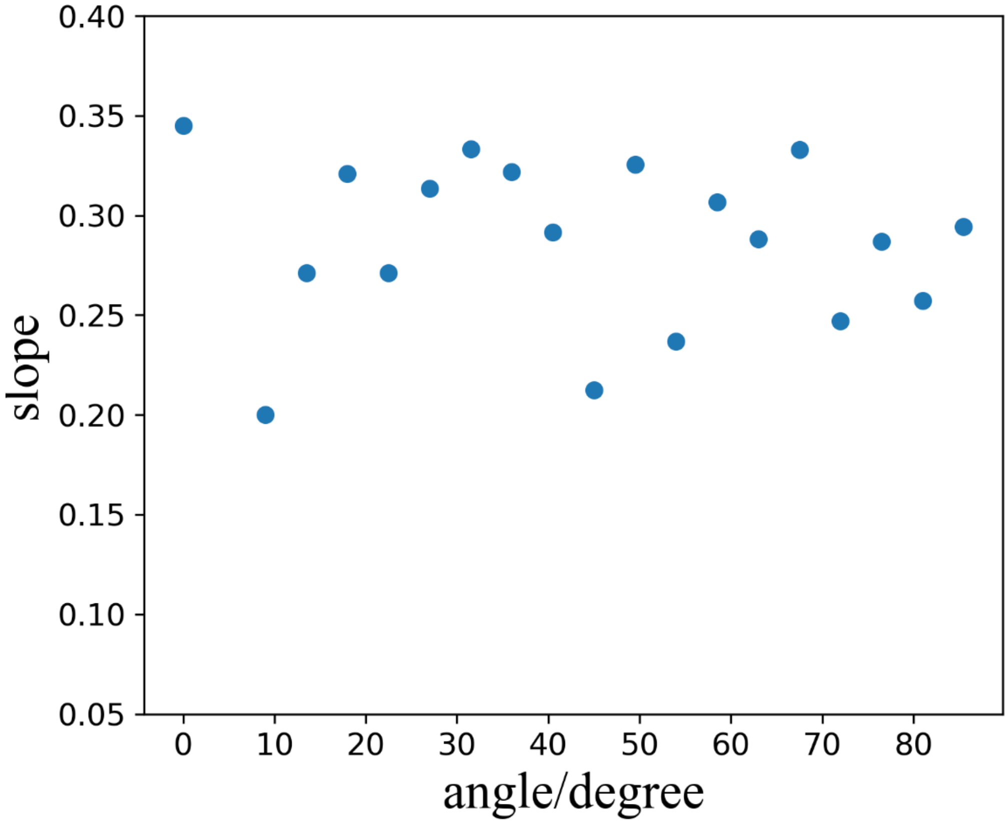

Supplementary Figure 7. KMC simulated coalescence slopes as a function of mismatch angles between two crystalline particles.
